# Heat-Induced Hatching of Red-Eyed Treefrog Embryos: Hydration and Clutch Structure Increase Behavioral Thermal Tolerance

**DOI:** 10.1093/iob/obac041

**Published:** 2022-09-28

**Authors:** Estefany Caroline Guevara-Molina, Fernando Ribeiro Gomes, Karen M Warkentin

**Affiliations:** Department of Physiology, Institute of Biosciences, Laboratory of Behavior and Evolutionary Physiology, University of São Paulo, São Paulo 05508-900, Brazil; Department of Physiology, Institute of Biosciences, Laboratory of Behavior and Evolutionary Physiology, University of São Paulo, São Paulo 05508-900, Brazil; Department of Biology, Boston University, Boston, MA 02215, USA; Smithsonian Tropical Research Institute, 0843-03092 Balboa, Panamá

## Abstract

Climate change is increasing both environmental temperatures and droughts. Many ectotherms respond behaviorally to heat, thereby avoiding damage from extreme temperatures. Within species, thermal tolerance varies with factors such as hydration as well as ontogenetic stage. Many tropical anurans lay terrestrial eggs, relying on environmental moisture for embryonic development. These eggs are vulnerable to dehydration, and embryos of some species can hatch prematurely to escape from drying eggs. Warmer temperatures can accelerate development and thus hatching, but excess heat can kill embryos. Thus, we hypothesize that embryos may show a behavioral thermal tolerance limit, hatching prematurely to avoid potentially lethal warming. If so, because warming and drying are often associated, we hypothesize this limit, measurable as a voluntary thermal maximum, may depend on hydration. We manipulated the hydration of the terrestrial eggs of *Agalychnis callidryas*, in intact clutches and egg-groups isolated from clutch jelly, then warmed them to assess if embryos hatch early as a behavioral response to high temperatures and whether their thermal tolerance varies with hydration or surrounding structure. We discovered that heating induces hatching; these embryos show a behavioral escape-hatching response that enables them to avoid potentially lethal warming. Hydrated eggs and clutches lost more water and warmed more slowly than dehydrated ones, indicating that hydration buffers embryos from environmental warming via evaporative cooling. Embryos in hydrated clutches tolerated greater warming before hatching and suffered higher mortality, suggesting their behavioral Thermal Safety Margin is small. In contrast, lower thermal tolerance protected dry embryos, and those isolated from clutch jelly, from lethal warming. Heat-induced hatching offers a convenient behavioral assay for the thermal tolerance of terrestrial anuran embryos and the interactive effects of warming and dehydration at an early life stage. This work expands the set of threats against which embryos use hatching in self-defense, creating new opportunities for comparative studies of thermal tolerance as well as integrative studies of self-defense mechanisms at the egg stage.

## Resumen

El cambio climático está aumentando tanto las temperaturas ambientales como las sequías. Muchos ectotermos responden conductualmente al calor, evitando así los daños por temperaturas extremas. Dentro de las especies, la tolerancia térmica varía con factores como la hidratación y la ontogenia. Muchos anuros tropicales depositan huevos terrestres que dependen de la humedad ambiental para el desarrollo embrionario. Estos huevos son vulnerables a la deshidratación y los embriones de algunas especies pueden eclosionar prematuramente para escapar de la desecación de los huevos. Las temperaturas más cálidas pueden acelerar el desarrollo y, por lo tanto, la eclosión, pero el exceso de calor puede matar a los embriones. Por lo tanto, planteamos la hipótesis de que los embriones pueden mostrar un límite de tolerancia térmica conductual, eclosionando prematuramente para evitar un calentamiento potencialmente letal. Si es así, debido a que el calentamiento y la desecación a menudo están asociados, planteamos la hipótesis de que este límite, medible como una tolerancia térmica voluntaria, puede depender de la hidratación. Manipulamos la hidratación de los huevos terrestres de *Agalychnis callidryas*, en posturas intactas y grupos de huevos aislados de la gelatina de la postura. Luego, los calentamos para evaluar si los embriones eclosionan temprano como respuesta conductual a las altas temperaturas y si su tolerancia térmica varía con la hidratación o la estructura circundante. Descubrimos que el calentamiento induce la eclosión. Estos embriones muestran una respuesta conductual de eclosión de escape que les permite evitar un calentamiento potencialmente letal. Los huevos y las posturas hidratadas perdieron más agua y se calentaron más lentamente que los deshidratados, lo que indica que la hidratación protege a los embriones del calentamiento ambiental a través del enfriamiento por evaporación. Los embriones en posturas hidratadas toleraron un mayor calentamiento antes de la eclosión y sufrieron una mayor mortalidad, lo que sugiere que su margen de seguridad térmica conductual es pequeño. En contraste, la tolerancia térmica más baja protegió a los embriones deshidratados, y a los aislados de la gelatina, del calentamiento letal. La eclosión inducida por calor ofrece un ensayo de comportamiento conveniente para la tolerancia térmica de los embriones terrestres de anuros y los efectos integrados del calentamiento y la deshidratación en una etapa temprana de la vida. Este estudio amplía el conjunto de amenazas contra las cuales los embriones usan la eclosión en defensa propia, creando nuevas oportunidades para estudios comparativos de tolerancia térmica, así como estudios integradores de mecanismos de autodefensa en etapa de huevo.

## Resumo

As mudanças climáticas estão aumentando as temperaturas ambientais e as secas. Muitos ectotermos respondem comportamentalmente ao calor, evitando danos devido a temperaturas extremas. Dentro das espécies, a tolerância térmica varia com fatores como a hidratação e a ontogenia. Muitos anuros tropicais depositam ovos terrestres que dependem da umidade ambiental para o desenvolvimento embrionário. Esses ovos são vulneráveis à desidratação, e os embriões de algumas espécies podem eclodir prematuramente para escapar da dessecação dos ovos. Temperaturas mais altas podem acelerar o desenvolvimento e, portanto, a eclosão, mas o excesso de calor pode matar os embriões. Portanto, propomos a hipótese de que os embriões podem mostrar um limite de tolerância térmica comportamental, eclodindo prematuramente para evitar o aquecimento potencialmente letal. Nesse caso, como o aquecimento e a dessecação são frequentemente associados, propomos a hipótese de que esse limite, mensurável como uma tolerância térmica voluntária, pode depender da hidratação. Manipulamos a hidratação dos ovos terrestres de *Agalychnis callidryas*, em desovas completas e grupos de ovos isolados da geleia da desova. Depois, aquecemos os ovos para avaliar se os embriões eclodem cedo como uma resposta comportamental a altas temperaturas, e se sua tolerância térmica varia com a hidratação ou a estrutura circundante. Descobrimos que o aquecimento induz a eclosão. Esses embriões mostram uma resposta comportamental de eclosão que lhes permite evitar o aquecimento potencialmente letal. Ovos e desovas hidratadas perderam mais água e se aqueceram mais lentamente que os desidratados, indicando que a hidratação protege os embriões do aquecimento ambiental através do resfriamento por evaporação. Os embriões em desovas hidratadas toleraram maior aquecimento antes da eclosão e sofreram maior mortalidade, sugerindo que sua margem de segurança térmica comportamental é menor. Por outro lado, a menor tolerância térmica protegeu os embriões desidratados e os isolados da geleia, do aquecimento letal. A eclosão induzida pelo calor oferece um teste comportamental conveniente para a estimativa da tolerância térmica de embriões de anuros terrestres e dos efeitos integrados do aquecimento e da desidratação em um estágio inicial da vida. Este estudo expande o conjunto de ameaças contra as quais os embriões usam a eclosão como defesa, criando novas oportunidades para estudos comparativos de tolerância térmica, além de estudos integrativos dos mecanismos de autodefesa no estágio de ovo.

## Introduction

Global warming is exposing organisms to changed climatic conditions, including higher environmental temperatures and more frequent droughts (less frequent rainfall) in some regions of the world (Allan and Soden 2008; Dai 2012; Trenberth et al. 2014). Anurans are particularly sensitive to these environmental changes due to their ectothermic condition and use of water bodies for reproduction (Lips et al. 2005; Pounds et al. 2006). Several studies show that increased environmental temperatures limit their dispersal capacity and influence their phenology, physiological functions, and behavior (Wygoda and Williams 1991; Navas et al. 2008, 2013; Parmesan 2007; Duarte et al. 2012). The effects associated with climate change interact with other factors such as emerging diseases (Pounds et al. 2006; Lips et al. 2008), invasive species (Miaud et al. 2016), and habitat loss (Schneider-Maunoury et al. 2016), increasing the extinction risk for anuran species (Stuart et al. 2004).

Tropical anuran species with terrestrial oviposition are among the most strongly affected by changes in climate because they deposit their eggs on land where they are exposed to high environmental temperatures and desiccation risk (Donnelly and Crump 1998; Mitchell 2002). While adults can avoid these conditions by changing body posture and seeking shelter and/or water to rehydrate (Wolcott and Wolcott 2001; Mitchell and Bergman 2016; Anderson and Andrade 2017), terrestrial egg clutches cannot move to avoid thermal stress or dehydration. During development, embryos facing high heat and/or dehydration may endure such conditions for some time, escape by hatching early, or die if these conditions persist and they cannot or do not hatch. While embryos themselves may exhibit some tolerance to heat and dehydration, the structural characteristics of clutches (e.g., well-hydrated jelly or foam surrounding eggs) may also play an important role to moderate or buffer these harsh conditions, at least for a time (Mendez-Narvaez et al. 2015).

Conditions outside the egg can impact the morphology, physiology, and biochemistry of the developing embryos (Mueller et al. 2015a,b; Mueller et al. 2019). For example, warmer environmental temperatures can increase the development rate of ectotherms (e.g., amphibians: McLaren and Cooley 1972; Kuramoto 1975; Harkey and Semlitsch 1988; Bradford 1990; Mitchell and Seymour 2000; Mueller et al. 2019) or, in worse cases, decrease hatching success (Ji and Du 2001; Mueller et al. 2011; Ojanguren and Brana 2003; Eme et al. 2015). However, we do not know if embryos can hatch early in response to heating, showing limits to behavioral thermal tolerance, evidenced as an escape response. One parameter to quantify such limits, the Voluntary Thermal Maximum (VT_Max_, Cowles and Bogert 1944), can be measured in warming experiments as the highest temperature an animal tolerates before moving away. Behavioral escape from heat enables animals to avoid exposure to temperatures closer to their physiological tolerance limit (measurable as their critical thermal maximum, CT_Max_). VT_Max_ provides a nonlethal method to estimate the vulnerability of ectotherms to stressful temperature conditions (Camacho et al. 2018). It has been studied in reptiles (lizards and snakes: Cowles and Bogert 1944; Cadena and Tattersall 2009; Díaz-Ricaurte and Serrano 2020, 2021; Díaz-Ricaurte et al. 2022b), and recently in adult anurans (Díaz-Ricaurte et al. 2020, 2022b; Guevara-Molina et al. 2020) and crickets (Díaz-Ricaurte et al. 2022a). The VT_Max_ can be affected by diel cycles, being lower at night than during the day (Díaz-Ricaurte et al. 2020), and in frogs (Guevara-Molina et al. 2020) the VT_Max_ is reduced in response to dehydration. Specifically, the decrease of VT_Max_ in response to dehydration shows an increase in Thermal Safety Margin to avoid reaching the physiological lethal limit of temperature, i.e., CT_Max_. The Thermal Safety Margin is used to estimate the difference between an organism's maximum physiological heat tolerance and the warmest temperature that it experiences (Sunday et al. 2014).

In embryos, drying-induced hatching is known from three frog clades that independently evolved terrestrial eggs, deposited in gelatinous egg masses on leaves above water, and from four species, including *Dendropsophus ebraccatus* (Touchon and Warkentin 2010; Touchon et al. 2011), *Hyalinobatrachium fleischmanni* (Delia et al. 2014), and two species of *Agalychnis* (*A. callidryas*, Salica et al. 2017; *A. spurrelli*, Gonzáles et al. 2021). However, we do not know whether these or any terrestrial frog embryos hatch early as a direct response to high temperatures, showing a behavioral thermal tolerance limit or, if so, whether dehydration alters this limit. Red-eyed treefrogs, *A. callidryas*, hatch early in response to multiple threats, including a fungal pathogen (Warkentin et al. 2001), predator attack (Warkentin 1995, 2000), and flooding (Warkentin 2002), as well as dehydration (Salica et al. 2017). This species’ robust escape-hatching response offers a tractable model for experimental studies of embryo behavior. Here, we used *A. callidryas* embryos to test for the expression of early hatching as an immediate behavioral response to high temperatures and to determine if the hydration level of eggs and egg-clutch jelly affects the response of embryos to warming. Our hypotheses were that: (1) embryos hatch early in response to warming (i.e., express a VT_Max_); (2) dehydration reduces the thermal tolerance of embryos; and (3) the natural clutch structure and materials associated with eggs increase the thermal tolerance of embryos.

## Materials and methods

### Clutch collection and hydration treatments

We conducted this research at the Smithsonian Tropical Research Institute (STRI) in Gamboa, Panama, during the 2018 and 2019 rainy seasons (June–August) with approval from the STRI Animal Care and Use Committee (2017-0601-2020-2) and research permits from the Panamanian Ministry of the Environment (SC/A-10-18 and SE/A-42-19). We collected clutches, on the leaves where they were laid, from STRI's Experimental Pond (9.120894 N, 79.704015 W; 45 m asl; WGS 84) when they were less than 1 day old. We took them to a laboratory at ambient temperature, where we duct-taped the leaves with clutches to plastic support cards and hung them over dechlorinated water in plastic cups. We randomly assigned clutches to wet and dry treatments and maintained them in covered, screened plastic boxes with hydration controlled at two levels. In the wet (hydration) treatment, clutches were automatically misted with rainwater every hour for 20 s using a MistKing system (www.mistking.com). With no external hydration, egg diameter decreases and embryos eventually die by drying (Salica et al. 2017). Therefore, in the dry (dehydration) treatment, clutches were monitored for excess dehydration three or four times daily and manually misted for 5 s when judged necessary for embryo survival (i.e., when the size of the eggs decreased to ≤4.0 mm diameter) (Salica et al. 2017). We maintained embryos in differential hydration treatments until age 5 days, midway through their plastic hatching period (age 3–7 days), when they are highly responsive to environmental cues and also highly discriminating, with few embryos hatching spontaneously (Warkentin 2017; Warkentin et al. 2017). We monitored developmental stages daily using a detailed staging table for *A. callidryas* (Warkentin 2017). We tested embryos’ responses to heating in two different contexts of the surrounding structure: in intact clutches (henceforth clutches) and in small groups of eggs isolated from jelly and leaf (henceforth egg-groups). Pairs of wet and dry clutches were set up at age 4 days, while eggs for paired wet and dry egg-groups were isolated from clutch jelly at age 3 days (see below). In both cases, embryos were tested for VT_Max_ at age 5 days (stages 32–34, Warkentin 2017). We attempted two warming trials per day, testing wet and dry treatments in random order starting at 8:00 am. In a few cases, a prepared clutch or egg-group hatched before we could test it, resulting in a single trial that day. Each sibship was used in only one trial.

### Heating clutches

**Fig. 1:**
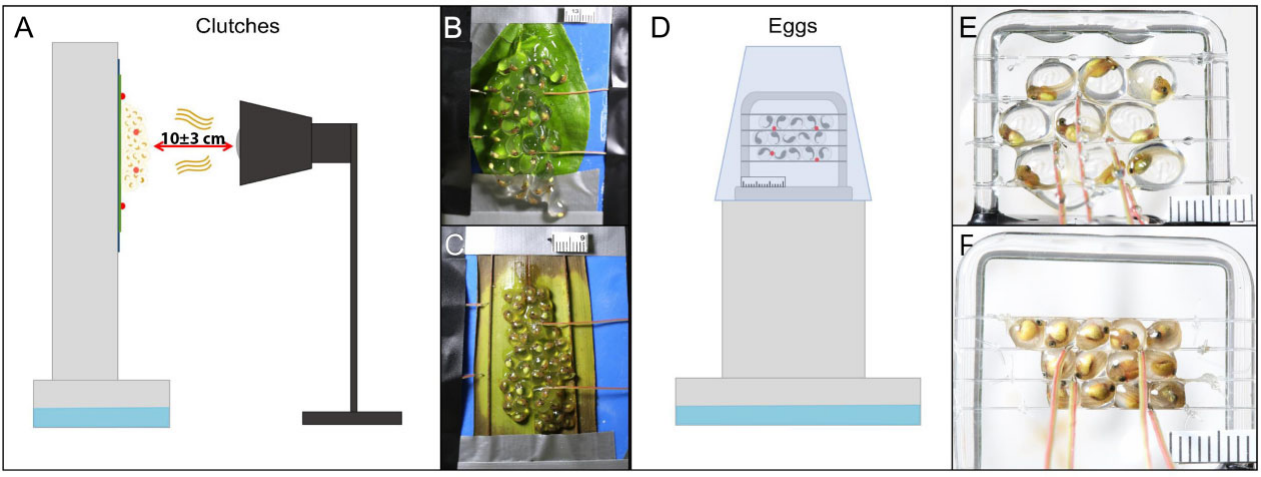
Set-up for testing embryos’ thermal tolerance in *Agalychnis callidryas*. (**A**) Side view of assembly for heating clutches, with thermocouple locations indicated by red dots. The clutch is on a support stand in a tray of water to catch tadpoles. (**B**) Fully hydrated and (**C**) dehydrated clutches, just before heating, showing scale and thermocouples. (**D**) Assembly for testing embryos’ thermal tolerance when isolated from the clutch structure, jelly, and leaf. The assembly is on a support stand over water to catch tadpoles covered with an inverted plastic cup to maintain humidity and egg size until the heating trial; dots (red in online version) indicate thermocouples. (**E**) Wet and (**F**) dry egg-groups, before heating.

We tested embryos’ responses to heating in six hydrated clutches and eight dehydrated clutches. We set up these clutches for experiments at age 4 days, after the onset of mechanosensory-cued hatching but before they became too difficult to handle without inducing hatching (Warkentin et al. 2017). We first checked for undeveloped (possibly unfertilized) eggs and, if possible, carefully removed them without altering the clutch structure. Then, we mounted each clutch on an individual rectangular support stand with a scale for subsequent measurements, placing two Omega Type T thermocouples inside the jelly, among the eggs, and two more touching the surface of the leaf. To minimize moving eggs on their test day, these assemblies were placed in a covered plastic box on the table where warming trials were conducted. The next day, for testing, we set an assembly in a tray of water, to catch hatchlings (Fig. 1A), and connected the thermocouples to a Pico Log TC-08 data logger, which was connected to a computer that recorded temperature every 10 s throughout the trial. We took initial digital photographs (Fig. 1B and C) of the clutch to measure egg size, using Image J (NIH) software, counted the number of eggs and measured the jelly thickness at the thickest point by inserting a fine probe orthogonally through the jelly, between the eggs, to the leaf surface. After this set-up procedure, we left embryos undisturbed for 5 min to allow for any mechanosensory-cued hatching, counting hatchlings or recounting embryos if necessary. We then observed embryos for 1 h at ambient temperature (mean 26.19°C ± 0.56 SD, range = 25.6–27.5°C, *N* = 14) to measure a baseline hatching rate. For each clutch, we subtracted its baseline hatching rate from the hatching rate measured during heating trials to calculate its heating-induced increase in hatching rate.

After recording the baseline hatching rate, we began heating the clutch using a rheostatically controlled 60 W ceramic bulb (Fig. 1A). Our goal was to heat clutches gradually and continuously without exposing the embryos to heat shock, thus preventing them from reaching their CT_Max_ quickly, following the dynamic heating method (Lutterschmidt and Hutchison 1997). We set initial bulb distance at 10 cm and rheostat setting at a moderate value based on pilot experiments, then continuously monitored the egg-temperature data, adjusting the distance (±3 cm) to limit the variation in heating rates. Specifically, if clutches warmed >0.5°C in a minute we increased the distance to reduce heat input, and if 3–4 min passed without an increase in temperature we reduced the distance to increase heat input. To prevent hatching tadpoles from contacting the bulb, the minimum clutch-to-bulb distance was 7 cm. However, wet clutches at this distance sometimes maintained a stable temperature, especially early in their trial; in this case, we increased the rheostat setting to achieve measurable warming. The mean egg-heating rate for each trial was calculated as the difference between initial and final mean temperatures (averaged across two thermocouples for clutches and four thermocouples for egg-groups) divided by trial duration. For clutches, we also calculated the mean heating rates of leaves using the difference between initial and final leaf temperatures (averaged across two thermocouples) divided by trial duration. We calculated individual mean heating rates for embryos as the difference between initial the mean egg temperature in the trial and the individual VT_Max_, divided by the duration of heating before the embryo hatched.

For each embryo hatched, we recorded its hatching time and the temperature of the nearest thermocouple. We stopped each heating trial when the clutch reached 40°C because the highest rainy season temperature recorded so far at the Experimental Pond is 39.90°C (Brandon Güell, *personal communication*) and exposure to 41°C was lethal to embryos in our pilot observations. After heating, we took a final photograph to count and measure the eggs remaining in the clutch, re-measured jelly thickness at the location of the original maximum thickness, and carefully removed the thermocouples. Undeveloped and visibly less-developed eggs, out of synchrony with their siblings, were not considered test subjects and excluded from all counts and measurements. At the end of experiments, we checked all remaining embryos for movement, heartbeat, or blood circulation in the external gills. We then used a blunt probe to prod and jiggle any motionless individuals lacking visible blood circulation, using methods that reliably induce mechanosensory-cued hatching (Warkentin et al. 2017); all were behaviorally unresponsive and considered to be dead. We monitored all hatchlings and any embryos that remained unhatched for 24 h to determine survival after heating, checking several times for signs of life and death as above. After 24 h, we jiggled any remaining live embryos to induce hatching and released all tadpoles at the Experimental Pond.

### Heating egg-groups

We tested embryos’ responses to heating using eggs from five hydrated clutches and seven dehydrated ones set up in isolation from their clutch jelly and leaf. We randomly assigned and applied hydration and dehydration protocols to whole clutches, as above, then removed eggs from their clutches just before the onset of mechanosensory-cued hatching at age 3 days, stages 25–27 (Warkentin 2017). For each trial, we removed 9–12 eggs from the same clutch, using forceps, and assembled them on a frame constructed from a glass arch mounted on a glass base, with monofilament nylon line stretched between the vertical posts at three levels to support the eggs (Fig. 1D and F). Eggs were thus in contact with each other in the plane of the frame but had greater air-exposure than in clutches at the front and back. Note that because dry eggs are smaller, the frame accommodated more of them (Fig. 1D and F). For each assembly, we distributed four Omega Type T thermocouples among the eggs to record temperature and placed a ruler at the base for scale (Fig. 1E and F). Each glass assembly with eggs and thermocouples was taped, for stability, to a plastic support stand and placed in a shallow tray of water to catch hatchlings. To maintain the desired egg size and hydration level, we covered the assemblies with an inverted plastic cup that was moistened inside to maintain high humidity (Fig. 1D). We monitored egg-groups and manually misted them with rainwater for 5 s three times at age 4 days. As above, we tested paired wet and dry egg-groups in random order each day, at age 5 days, but in some cases only one assembly had sufficient unhatched eggs to test (criteria: at least 8 wet eggs or 10 dry eggs). For heating trials, we removed the plastic cups, counted the eggs, and connected the thermocouples to the datalogger and computer for recording, then waited for five undisturbed minutes to allow for any mechanosensory-cued hatching as above. We positioned a Canon EOS 5D camera with an EF 100 mm macro lens on a tripod in front of the assembly with a time-lapse controller set to photograph the eggs and scale every 2 min. We first observed the embryos for 1 h at ambient temperature (mean 26.29°C ± 0.70 SD, range = 24.9–27.8°C, *N* = 12) to measure a baseline hatching rate, then heated the assemblies with a 60 W ceramic bulb located behind the eggs. Based on pilot experiments, we began trials at a moderate rheostat setting and a 15 cm egg-to-bulb distance. We continuously monitored temperature data, adjusting the distance (±5 cm) and, if need be, the rheostat to limit variations in heating rate as above. When an embryo hatched, we recorded its hatching time and the temperature of the nearest thermocouple. Trials ended when all embryos hatched or the temperature reached 40°C, as above. We checked for mortality and monitored all test subjects for 24 h after trials, as above, then released the tadpoles, as well as their siblings that remained in the clutch and were not used in heating trials, at the Experimental Pond.

### Egg-volume measurements

To assess evaporative water loss, we used ImageJ (NIH) to measure egg diameters from initial and final clutch and egg photographs, then calculated egg volumes based on spherical geometry, using the formula *V* = (4/3) π r^3^. For clutches, we measured up to 20 randomly selected, fully visible eggs in the initial photo. Some eggs were partially obscured behind siblings, thus not initially measurable, but became visible when siblings hatched. We measured all fully visible, unhatched eggs in the final photo (never >20). Thus, initial and final measurements from clutches are of only partly overlapping subsets of eggs, and sample sizes are smaller for final measurements. For egg-groups, we measured every egg in the initial photo, then used the last pre-hatching photo in which an egg appeared intact to measure its final size. Thus, we obtained final sizes for more eggs, but at different times depending on when embryos hatched. An analysis of egg-volume loss rates indicated that our final measurements for a few individuals (*N* = 7 outliers, from three dry egg-groups) likely occurred after the embryos had already ruptured their eggs, rapidly losing volume in the hatching process. We therefore excluded these individuals from our analysis of evaporative volume loss.

### Statistical analysis

To assess hydration treatment effectiveness, we compared egg size (diameter) between dry and wet treatments separately for clutches and for egg-groups using *t*-tests. We also compared jelly thickness (for clutches) and heating trial durations across hydration treatments, within structures, using *t*-tests. We used linear models to test for effects of hydration (dry and wet) and structure (clutches and egg-groups) on heating-induced increase in hatching rate, hatching temperature (VT_Max_), and changes in egg volume during heating. For hatching-rate increase and egg volume, initial residual plots revealed heteroscedasticity, so we log-transformed the data to correct this for the final analysis. Shapiro–Wilk tests determined that response variables (log-hatching rate increase, VT_Max_, log-egg-volume) were normally distributed. For VT_Max_ and egg volume, we fitted linear mixed effects models (lme4 package, “lmer” function; Bates et al. 2015), including sibship (trial) identity as a random factor to account for multiple measurements within a trial. This random factor was never significant, therefore we pooled eggs across replicates for graphical representation of data. For egg volume, we included time-point (coded as a factor with two levels, initial and final) to assess changes across the heating trial; because of the possible large number of interactions with three fixed factors, we used the Akaike Information Criterion (AIC; Akaike 1973; Bozdogan 2000) to select the best fit model, based on the lowest AIC value (Wang and Qun 2006).

Neither trial-mean heating rates nor embryo mortality rates (proportion that died during heating) met parametric assumptions. Therefore, we used Kruskal–Wallis tests to compare across our four hydration × structure categories (i.e., wet and dry clutches and wet and dry egg-groups), then used Wilcoxon tests for pairwise comparisons between categories. To compare the heating rates of leaves and the clutches attached to them, we applied paired-samples Wilcoxon tests to data from wet and dry treatments separately. Because trial-mean heating rates varied across hydration × structure categories, we also assessed the effects of individual egg-heating rates on the VT_Max_ of individuals by including it as a covariate in a linear mixed effects model along with structure and hydration as fixed factors and the random factor of sibship. Here, because of the many possible interactions between the covariate and fixed factors, we also used an AIC-based model-selection approach to choose the best model. Finally, to assess if variation in heating rate might account for the effects of hydration and structure in our initial analysis of VT_Max_, we repeated that analysis on a restricted dataset, excluding all individuals with heating rates over 0.2°C/min (61 individuals from 3 clutches and 4 egg-groups, including all individuals in the dry clutch with the highest heating rate). All analyses were performed in R V. 4.0.2 (R Core Team 2020) and plotted in Sigmaplot version 14.5 from Systat Software, Inc., San Jose, CA, USA, www.systatsoftware.com.

## Results

### Hydration treatment effectiveness

Our treatments successfully generated differences between wet and dry eggs and clutches. At the start of trials, eggs were smaller in dry than in wet clutches (*t* = −13.52, df = 12, *P* < 0.001; Table 1) and smaller in dry than in wet egg-groups (*t* = −9.71, df = 10, *P* < 0.001; Table 1). In addition, the maximum jelly thickness was greater in wet than in dry clutches (*t* = −6.399, df = 12, *P* < 0.001; Table 1). More details can be found in Supplementary data, Table S1.

**Table 1:** Differences between wet and dry *Agalychnis callidryas* in intact clutches on leaves and in egg groups isolated from jelly and leaf.

	Structure	Hydration	Mean ± SD	Range	*N*
Initial egg diameter (mm)	Clutches	Wet	6.43 ± 0.53	5.21 − 7.47	110
		Dry	4.14 ± 0.42	2.78 − 4.70	147
	Egg-groups	Wet	6.10 ± 0.55	5.01 − 7.46	45
		Dry	3.93 ± 0.35	3.07 − 4.95	85
Jelly thickness (mm)	Clutches	Wet	7.66 ± 2.06	5.0 − 10	6
		Dry	2.06 ± 1.20	0.5 − 4.0	8
Baseline hatching rate (% embryos/h)	Clutches	Wet	0.00 ± 0.00	0.00 − 0.00	6
		Dry	1.65 ± 1.64	0.00 − 5.00	8
	Egg-groups	Wet	0.00 ± 0.00	0.00 − 0.00	5
		Dry	1.39 ± 2.31	0.00 − 8.33	7
Hatching rate during heating (% embryos/h)	Clutches	Wet	8.58 ± 2.57	4.71 − 13.22	6
		Dry	43.40 ± 24.31	21.88 − 109.09	8
	Egg-groups	Wet	21.77 ± 1.11	20.62 − 23.26	5
		Dry	66.13 ± 24.01	34.48 − 107.14	7
Heating trial duration (min)	Clutches	Wet	286 ± 68.23	197 − 391	6
		Dry	146 ± 65.42	55 − 229	8
	Egg-groups	Wet	276 ± 16.04	258 − 291	5
		Dry	107 ± 45.18	56 − 174	7
Mean heating rates (°C/min)	Clutches	Wet	0.04 ± 0.01	0.03 − 0.06	6
		Dry	0.10 ± 0.05	0.05 − 0.19	8
	Leaves	Wet	0.09 ± 0.02	0.06 − 0.13	6
		Dry	0.18 ± 0.09	0.09 − 0.34	8
	Egg-groups	Wet	0.02 ± 0.00	0.02 − 0.02	5
		Dry	0.06 ± 0.04	0.02 − 0.12	7
Voluntary thermal maximum (°C)	Clutches	Wet	37.77 ± 0.88	36.12 − 39.83	80
		Dry	36.13 ± 1.69	29.91 − 39.28	225
	Egg-groups	Wet	34.21 ± 0.58	33.14 − 35.48	45
		Dry	31.37 ± 0.92	28.92 − 32.93	85

### Heating trial durations

The mean duration of trials was shorter for dehydrated clutches and longer for hydrated clutches (*t* = −3.86, df = 12, *P* = 0.002; Table 1). Only two clutches, both dry, had complete hatching before reaching 40°C. Similarly, the mean duration of trials was shorter for dry egg-groups and longer for wet egg-groups (*t* = −7.95, df = 10, *P* < 0.001; Table 1, Supplementary data, Table S1). In all trials with egg-groups, all embryos had hatched before reaching 40°C, except for one individual that died.

### Hatching rate

**Fig. 2:**
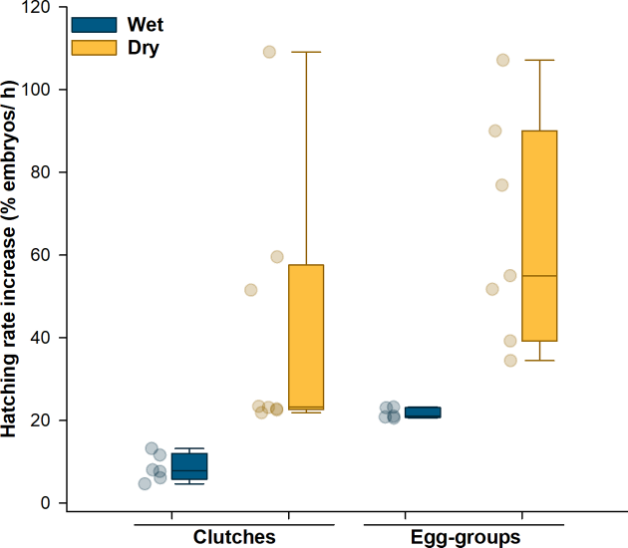
Heating-induced increase in the hatching rate of *Agalychnis callidryas* for wet and dry embryos (dark and light bars, or blue and yellow online, respectively) in two structures (clutches and egg-groups). Data points show the increase in hatching rate for individual clutches or egg-groups, above their baseline values, jittered for visibility; box plots show the median and first and third quartiles, and whiskers show the 5th and 95th percentiles of the data.

Hydrated eggs in both structures had a baseline hatching rate of zero at ambient temperature (Table 1) and, across structures, dehydration elevated the baseline hatching rate (Wilcoxon rank sum test: *X^2^* = 4.30, df = 1, *P* = 0.038; Table 1). Across every context of hydration × structure, heating increased hatching from baseline levels (Fig. 2 and Table 1). Hydration and structure both affected the extent to which hatching rate increased under heating, with no significant interaction (Fig. 2; Table 2 and Supplementary data, Table S1). The heat-induced increase in hatching rate was much greater for dry than for wet eggs, and somewhat greater for eggs removed from their jelly and leaf than for eggs in their natural clutch structure (Fig. 2; Table 2).

**Table 2:** Analyses of the heating-induced increase in hatching rate, hatching temperature of embryos (VT_Max_), and variation in egg volume for *Agalychnis callidryas* embryos under different conditions of hydration (wet, dry) and structure (clutches and egg-groups) and, for egg volume, at two time-points (initial and final measurements)

	Values	Std. error	*t*-value	*P*-value
Hatching rate increase:				
Intercept	3.543	0.160	22.182	2.0E–16***
Hydration	−1.455	0.244	−5.963	5.31E–06***
Structure	0.554	0.234	2.369	0.027*
Hydration × structure	0.437	0.360	1.214	0.237
Voluntary thermal maximum:				
Intercept	36.280	0.236	153.745	2.0E–16***
Hydration	1.560	0.376	4.154	0.0003***
Structure	−4.896	0.358	−13.677	1.8E–12***
Hydration × structure	1.269	0.568	2.233	0.0344*
Egg volume:				
Intercept	3.540	0.057	62.385	2E–16***
Time-point	−0.447	0.033	−13.572	2E–16***
Hydration	1.301	0.088	14.854	2.94E–14***
Time-point × hydration	−0.436	0.049	−8.914	2E–16***

* *P* < 0.05

*** *P* < 0.001

### Heating rates

**Fig. 3:**
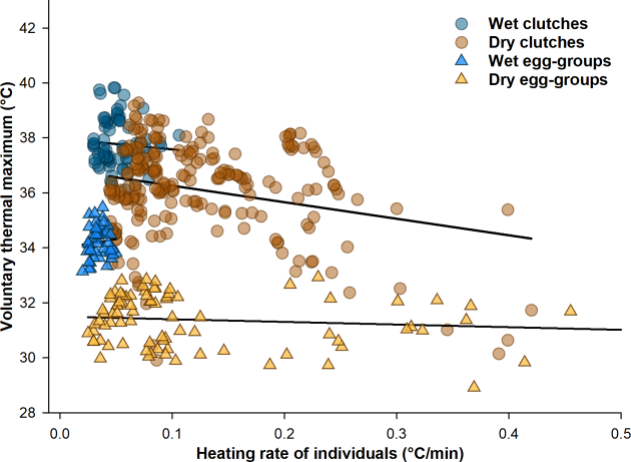
Relationship between the hatching temperatures of embryos (VT_Max_) and their individual egg-heating rates. Circles represent embryos in clutches and triangles represent those in egg-groups, with hydration status color-coded (dark/blue for wet, light/brown for dry).

**Fig. 4:**
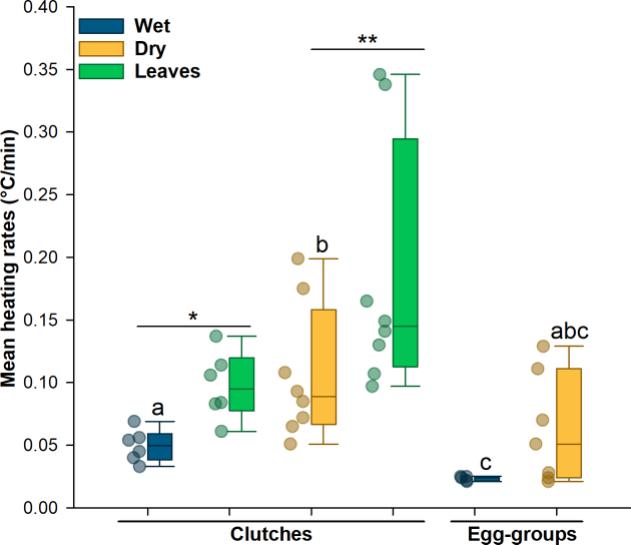
Mean heating rates for wet and dry clutches, the leaves to which they were attached, and wet and dry egg-groups. Data points are mean heating rates calculated across each trial; box plots show the median and first and third quartiles, and whiskers show the 5th and 95th percentiles of the data. Different letters indicate significantly different egg-heating rates across contexts (all *P* < 0.05); asterisks indicate different heating rates of leaves and their attached clutches (*P < 0.05, **P < 0.01).

The mean heating rates differed among structure-hydration categories (Kruskal–Wallis test: *X^2^* = 13.13, df = 3, *P* = 0.004; Fig. 3; Table 1 and Supplementary data, Table S1). Dry clutches had higher heating rates than wet clutches (*P* = 0.012) and wet egg-groups (*P* = 0.004), and wet egg-groups also warmed slower than wet clutches (*P* = 0.008). However, heating rates of dry egg-groups were similar to those of wet egg-groups and wet and dry clutches (all *P* values > 0.1; Fig. 3; Table 1). Comparing the heating rates of leaves to clutches, wet leaves warmed faster than their attached clutches (*V* = 21, *P* = 0.031; Fig. 4; Table 1) and dry leaves warmed much faster than their attached clutches (*V* = 36, *P* = 0.007; Fig. 4; Table 1).

### Voluntary thermal maximum

**Fig. 5:**
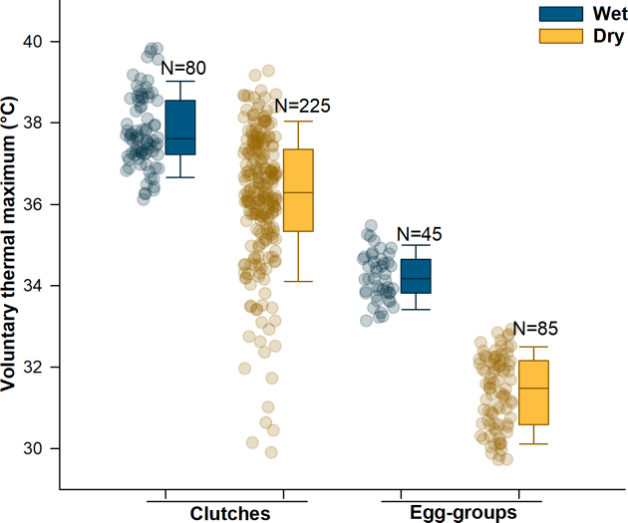
Voluntary thermal maximum (VT_Max_) showing limits to behavioral thermal tolerance of *Agalychnis callidryas* embryos. VT_Max_ values are the last recorded temperatures from the nearest thermocouple, just before embryos hatched during heating in two structures (clutches and egg-groups), following development in two hydration treatments (dark/blue indicates wet, light/yellow dry). Data points are values for individual embryos; box plots show the median and first and third quartiles, and whiskers show the 5th and 95th percentiles of the data.

Hydration, structure, and their interactions all significantly affected the temperature at which embryos hatched (Fig. 5; Table 2). Embryos in clutches expressed higher VT_Max_ than did embryos in egg-groups, and in both structures, wet embryos had a higher VT_Max_ than dry embryos (Fig. 5; Table 1 and Supplementary data, Table S2). However, the effect of hydration on VT_Max_ was weaker in clutches, with substantial variation within the dry treatment and overlap across hydration levels; the effect of hydration was stronger for egg-groups (Fig. 5). All embryos that hatched during heating trials successfully survived 24 h after exposure; the tadpoles were mobile and appeared healthy. Moreover, there was no post-trial mortality of embryos that remained unhatched; the only mortality was of individuals that died *in ovo* during heating.

Because trial-mean heating rates varied among structure-hydration categories, we also analyzed embryo VT_Max_ with individual egg-heating rate included as a covariate (Fig. 3; Table 3). The best model included all main effects and interactions, with significant effects of heating rate, structure, and a heating-rate by structure interaction; the effect of hydration was not significant (AIC = 1273.79; 22.37 units below the next best model with AIC = 1296.16; Table 3).

**Table 3:** Best fit model to explain hatching temperatures (VT_Max_) of individual embryos based on individual egg-heating rates (as a covariate), hydration (wet and dry), structure (clutch and egg-groups), their interactions, and the random factor of sibship.

	Values	Std. error	*t*-value	*P*-value
Intercept	39.2053	0.4817	81.392	2E–16***
Heating rate	−22.714	1.5847	−14.33	2E–16***
Hydration	0.6454	1.0747	0.601	0.55
Structure	−7.6771	0.692	−11.09	2.12E–11***
Heating rate × hydration	−13.766	14.9849	−0.919	0.359
Heating rate × structure	21.652	1.955	11.075	2E–16***
Hydration × structure	1.749	1.5446	1.132	0.261
Heating rate × hydration × structure	23.2289	27.932	0.832	0.406

*** *P* < 0.001

When individuals showing the highest heating rates (>0.2°C/min) were excluded from analysis, VT_Max_ was still affected by hydration, structure, and their interaction in a model without heating rate, and by structure, heating rate and their interaction, but not by hydration, in a model including heating rate as a covariate (Supplementary data, Table S3). Thus, analyses excluding individuals with the highest heating rates, which occurred only in dry treatments, mirrored those of the complete data set (compare Tables 2 and 3 and Supplementary data, Table S3), indicating that the substitution of hydration by heating rate as explanatory variables for VT_Max_ does not depend on extreme heating rate values.

### Egg-volume loss

**Fig. 6:**
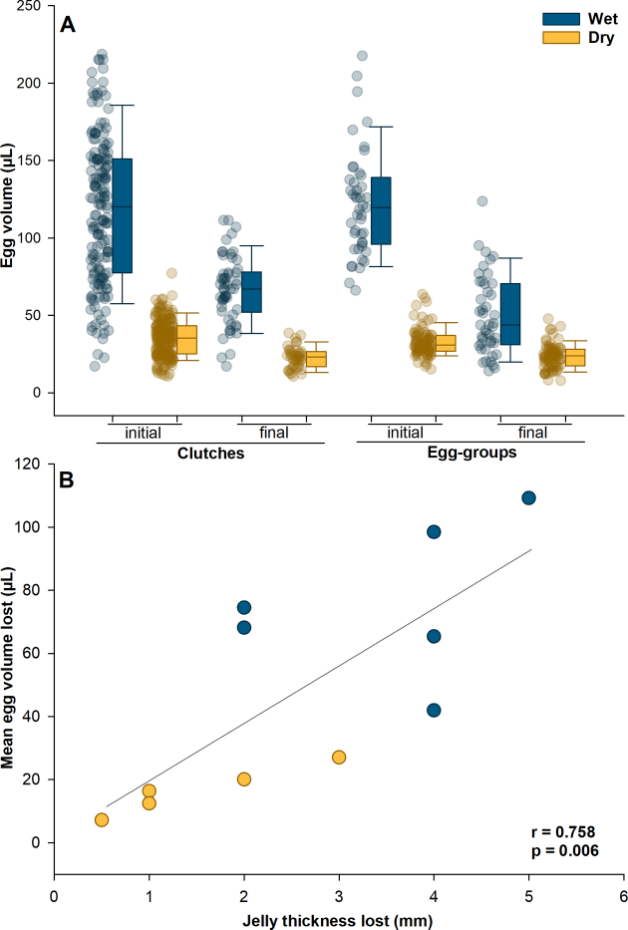
(**A**) Initial pre-heating and final post-heating volumes of *Agalychnis callidryas* eggs heated in two structures (clutches and egg-groups), following development in two hydration treatments (dark/blue indicates wet, light/yellow dry). Clutch data include all eggs measured at the start of the trial (*N* = 110 wet, 147 dry) and the end of the trial (54 wet, 41 dry). Egg-group data include all individuals tested at the start of the trial (*N* = 45 wet, 85 dry) and final measurements for most of the same individuals just before each hatched (*N* = 45 wet, 78 dry). Data points are values for individual eggs; box plots show the median and first and third quartiles, and whiskers show the 5th and 95th percentiles of the data. (**B**) Correlation between the mean egg-volume lost and the clutch thickness lost across all clutches measured.

The egg volume was significantly affected by time-point, hydration, and the time-point by hydration interaction, but the best model included no main or interaction effects of structure (AIC = 259.05; 2.65 units below the next best model with AIC = 261.70; Table 2 and Supplementary data, Table S2). During heating, large well-hydrated eggs, both in intact clutches and in egg-groups isolated from jelly, lost more volume than dry eggs, which started out smaller (Fig. 6A). Clutches lost volume from both eggs and jelly (Table 1), and the mean egg-volume lost was closely correlated with the jelly thickness lost (Pearson correlation: *r* = 0.75, *P* = 0.006; Fig. 6B).

### Embryo mortality during heating

**Fig. 7:**
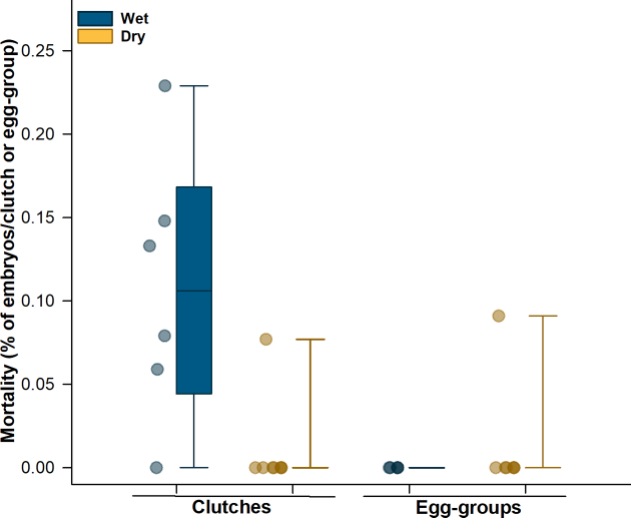
Mortality of embryos during heating trials in clutches and egg-groups. All mortality occurred *in ovo* during heating; all hatched tadpoles and all live embryos that remained unhatched at the end of heating survived for 24 h after testing. Data points are values for each clutch or egg-group; box plots show the median and first and third quartiles, and whiskers show the 5th and 95th percentiles of the data.

Embryo mortality differed among structure-hydration categories (Kruskal–Wallis test: *X^2^* = 13.10, df = 3, *P* = 0.004; Fig. 7). Wet clutches had greater mortality compared to dry clutches (*P* = 0.010), wet egg-groups (*P* = 0.017) and dry egg-groups (*P* = 0.024), none of which differed from each other (all *P* values ≥ 0.5). Based on an examination of end-of-trial photographs, the embryos that died in wet clutches all had large amounts of perivitelline fluid remaining. Embryos that died in dry treatments (one clutch and one egg-group), while much more spatially constrained, still had sufficient egg volume to allow position changes. There were no obvious differences between embryos that died and their siblings that survived unhatched.

## Discussion

We discovered that *A. callidryas* embryos hatch early as a rapid, behavioral response to heating, and this escape behavior enables them to avoid lethal egg temperatures. We also found that embryos’ microenvironmental context, including hydration and clutch structure, affects both the risk of warming to which they are exposed and their behavioral thermal tolerance. These results expand our understanding of the vulnerability and self-defense mechanisms of terrestrial anuran embryos facing hydric and thermal stresses.

In addition to temperature, terrestrial frog embryos are sensitive to dehydration (Salica et al. 2017; Rudin-Bitterli et al. 2020). The evidence that drying changes thermal tolerance indicates that our understanding of potential general patterns in the thermal sensitivity of terrestrial anuran embryos is severely limited. Such information may be especially important for terrestrial-breeding tropical species, whose eggs are considered particularly vulnerable to climate change (von May et al. 2017; Hoffmann et al. 2021). Moreover, while hatching is well-documented as an embryo defense against egg predators, pathogens, and the abiotic threats of hypoxia and dehydration (Warkentin 2011a, b), to our knowledge, our study is the first to investigate if hatching occurs as an embryo response to potentially lethal warming.

### Effect of hydration on hatching temperature

Warming and drying conditions are typically associated; rainfall both lowers temperatures and provides hydration, and high temperatures increase dehydration risk, especially for egg clutches on land (Méndez-Narváez et al. 2015). Thus, it is important to consider the effects of temperature in a hydric context, and of hydration in a thermal context, to understand both the eco-physiology of embryos and their potential for adaptive behavioral responses. We found that *A. callidryas* embryos hatched at lower temperatures and in less time when their eggs were drier; that is, their VT_Max_ decreased under dehydration. This was evident for embryos in intact clutches and particularly clear for eggs removed from their clutch jelly and the leaf on which they were laid.

Dehydration also decreases VT_Max_ in juvenile anurans, and this effect is stronger than the effect of drying on CT_Max_, suggesting that dry frogs maintain a greater Thermal Safety Margin (Guevara-Molina et al. 2020). The reduction in embryo VT_Max_ with egg dehydration might, similarly, reflect a change in Thermal Safety Margin. However, we do not know if or how hydration affects the temperature that is lethal to embryos or how temperature affects the level of dehydration that is lethal. To further understand variation in the Thermal Safety Margin of *A. callidryas* embryos, it will be necessary to determine dangerous levels of dehydration and temperature (i.e., CT_Max_) to compare with our VT_Max_-hydration data. Moreover, the effect of hydration on VT_Max_ was weaker in clutches, with substantial variation within the dry treatment and overlap across hydration levels (see Fig. 5); in contrast, embryos in wet and dry egg-groups showed distinctly different VT_Max_. This difference suggests that the clutch structure and/or oviposition substrate (jelly + leaf) reduce the effect of hydration level on embryo thermal tolerance, but this buffering varies substantially under dry conditions.

### Effect of heating rate on hatching temperature

Heating rates differed across structure-hydration categories (Figs. 3 and 4) and including heating rate as a covariate (Table 3) revealed its influence on VT_Max_ in both main and interaction effects, replacing the effect of hydration. Analyses excluding individuals with the highest heating rates, which occurred only in dry treatments, mirrored those of the complete data set (compare Tables 2 and 3 and Supplementary data, Table S3), indicating that the exchange of heating rate for hydration as an explanatory variable for VT_Max_ does not depend on these extreme values. Thus, at least part of the variation in thermal tolerance in our warming trials seems attributable to an effect of heating rate on VT_Max_. In particular, higher heating rates were associated with lower VT_Max_, and the embryos in dry clutches and egg-groups, which had higher heating rates, had lower VT_Max_ compared to those in wet clutches and egg-groups (Figs. 3–5). While we do not yet know how these embryos sense temperature or process that information for hatching decisions, it is possible that heating rate interacts with these underlying mechanisms to alter behavioral thermal tolerance (Tattersall et al. 2012).

Other studies have found that heating rates can affect ectotherms’ thermal tolerances, but the existence and direction of these effects vary. For instance, in bullfrogs, *Lithobates catesbeianus*, higher heating rates do not alter VT_Max_, although they do increase CT_Max_ (Guevara-Molina et al. 2020). In two foam frogs (*Physalaemus cuvieri* and *P. nattereri*; Díaz-Ricaurte et al. 2020) and a snake (*Bothrops pauloensis*; Díaz-Ricaurte and Serrano 2021), higher heating rates are associated with higher VT_Max_. For *A. callidryas* embryos, experimentally separating the effects of heating rate and hydration will require better control of heating rate across hydration levels. Nonetheless, at similar low heating rates, where *A. callidryas* embryos in clutches showed the most overlap in VT_Max_, embryos in egg-groups still showed distinctly lower VT_Max_ when dry (Fig. 3). This is evident statistically in the interaction effect and suggests some role of hydration, *per se*, in changing embryo thermal tolerance. The fact that drying alone, without warming, can induce early hatching in *A. callidryas* is well-documented (Salica et al. 2017; Tippett and Warkentin 2017) and also evident in our baseline hatching rates (see Table 1). The mechanism underlying this response is unknown but might involve increased osmolality of perivitelline and body fluids or the increased concentration of specific molecules, such as ammonia (Méndez-Narvaez and Warkentin 2022). In addition, these embryos are known to combine information across cue properties and across sensory modalities for their hatching decisions (Warkentin and Caldwell 2009; Güell and Warkentin 2018; Jung et al. 2020). Thus, it is certainly plausible that embryos adjust their VT_Max_ in direct response to their hydration state.

### Structure, hydration, and heating rate

The clutch structure (e.g., hydrated jelly) and oviposition site provided by parents can directly influence embryos’ susceptibility to environmental threats, including drying and predation (Touchon and Warkentin 2009; Delia et al. 2017, 2020). They also influence warming. First, dehydration substantially reduced both jelly thickness and egg diameter, as reported previously for *A. callidryas* (Salica et al. 2017). In this species, females deposit water into the jelly at oviposition, enabling the initial expansion of the perivitelline space (Salthe 1965; Pyburn 1970; Salica et al. 2017), and further egg and jelly hydration depends on rainfall. The large eggs and thick jelly core of well-hydrated clutches provide both a hydric reserve and a thermal buffer for embryos. Under our testing conditions, it was surprisingly hard to warm up wet clutches and egg-groups, and also difficult to avoid warming dry clutches and egg-groups too quickly. Although the need to adjust heat input to reduce this variation in warming rates in itself reveals differences in thermophysics, it also precludes accurate empirical assessment of their resistance to warming. Measuring warming rates under equal heating could clarify the magnitude of the thermal buffering effects provided by clutch structure and hydration.

There are two potential mechanisms by which hydration could slow warming: adding water (1) increases the thermal mass and so increases the energy required for a given temperature increase, and (2) increases the amount of evaporative cooling that can occur by water loss. If we assume that the specific heat of eggs falls between freshwater and seawater (4.18 and 3.99 J/gC, respectively, at 30°C), then based simply on their average sizes, we estimate that wet eggs require about 3.7 times as much energy per unit of warming than as dry ones (ca. 5.61 J versus 1.51 J, respectively, for a 10°C increase). Considering that dry eggs have higher osmolality and density (i.e., closer to seawater), they may take even less energy to warm (ca. 1.47 J for 10°C), magnifying the difference. Similarly, based on the average egg volume lost (76 versus 14 µL), we estimate that wet eggs lost about 5.6 times more heat by evaporation than did dry eggs. Based on the heat of vaporization for freshwater, this would be ca. 185 versus 33 J, although the higher density of dry eggs may reduce their evaporative heat loss slightly (ca. 32 J). Thus, the effects of evaporative cooling on egg warming appear both absolutely and relatively larger than those of thermal mass. Even with greater heat input, our results indicate that volume loss from wet eggs and hydrated jelly slowed temperature rise, and the dry eggs heated up faster. This suggests that hydrated eggs and intact gelatinous clutches have substantial thermal buffering capacity via evaporative cooling.

### Survival and mortality during heating

All embryos that hatched during heating successfully survived 24 h after exposure, indicating that tadpole viability was not compromised by the heating they experienced before hatching or by the heat-induced hatching process. Thus, heat-induced hatching is an effective way for embryos to escape from a dangerously warming microenvironment. In a warming climate, some embryos may exceed their optimal and indeed their viable thermal range, with little or no capacity to avoid harm or death. Yet others, of some species and in later developmental stages, may hatch early to avoid overheating. Testing the effects of experimental warming on the behavior of ectotherms in late embryonic stages could help to elucidate their thermal tolerances and vulnerabilities under changing environmental conditions (Collin et al. 2021; Vorsatz et al. 2021).

Our pilot observations indicated that a temperature of 41°C was lethal for *A. callidryas* embryos, so we ceased warming eggs at 40°C. Nonetheless, some embryos did not hatch and were dead at the end of their heating trial, while their siblings—both hatched and *in ovo*—survived. In the two dry replicates (one clutch and one egg-group) where embryos died, eggs still appeared large enough to allow survival, and across replicates, other embryos in similar-sized eggs survived. However, only 4 of 375 dry embryos (1.07%) died during warming in dry treatments, suggesting this is a relatively rare event. Because it is substantially more difficult for embryos to exit from dry versus wet eggs (Tippett and Warkentin 2017), it is also possible that this dry-treatment mortality represents or includes failed hatching attempts. If so, with 4 deaths and 312 embryos successfully hatched, then the failure rate is also low (≤1.27%).

Most of the embryos that died during heating were in wet clutches (21 of 197 test individuals, across 5 of 6 clutches). On average, 10.8 ± 0.1% of embryos died in this treatment, while 40.7 ± 18.6% hatched and 48.5 ± 17.0% remained alive *in ovo*. The fact that so many embryos in wet clutches, which easily could have hatched, instead died *in ovo* during heating suggests these embryos failed to perceive that they were in danger. This difference in response, compared to dry embryos, may reflect the general association of warming with drying and historical rarity of wet-hot conditions, which may have made embryos less adapted to recognize them. These findings also suggest that embryos may use a combination of cues to hatch (i.e., temperature and hydration state).

However, the difference between wet clutches and wet egg-groups, where there was 100% hatching and no mortality, suggests that factors in addition to heat and hydration modulated hatching decisions and mortality risk. The key difference between clutches and egg-groups was structure. In clutches, eggs were surrounded by siblings in the plane of the clutch, backed by jelly and leaf, and air-exposed only at the front, toward the heat source. In egg-groups, they were surrounded by siblings in the plane of the frame and exposed to air both at the front and back, toward and away from the heat source. Oxygen availability and uptake capacity can limit thermal tolerance, and embryos may be particularly strongly affected (Frederich and Pörtner 2000; Smith et al. 2015; Vorsatz et al. 2021). There are strong oxygen gradients within *A. callidryas* eggs, and, to maintain high oxygen uptake, embryos typically position their external gills in the high-oxygen region at the air-exposed surface (Warkentin et al. 2005; Rogge and Warkentin 2008). This air-facing orientation also ensures that embryos will hatch into the air and can fall to the water rather than hatching into the jelly where they can be trapped between their siblings and the leaf (Güell and Warkentin 2018). In addition to orienting in oxygen gradients (Rogge and Warkentin 2008), *A. callidryas* embryos may, like turtle embryos (Zhao et al. 2013; Ye et al. 2021), position themselves in thermal gradients within their eggs. We observed that as embryos were heated, especially wet ones, they moved more frequently inside the egg, while those in dry eggs seemed to spend more time stationary. Video recordings of embryos during VT_Max_ tests would allow quantification of their activity under different conditions. In our clutch warming set-up, embryos moving away from the heat would also move away from the oxygen, and into a position from which they should not hatch. This behavioral conflict between oxygen-oriented and potentially heat-oriented positioning *in ovo* may have delayed hatching. It may also have increased the risk of embryo mortality via a combination of thermally elevated oxygen demand and reduced oxygen supply, as embryos spent more time away from the air-exposed egg surface. Embryos in egg-groups, in contrast, had two air-exposed patches of egg-surface, one toward and one away from the heat source, alleviating any potential behavioral conflict or elevated risk. Future work should test the hypothesis that, before hatching, embryos first attempt to move away from heat within their eggs. If so, then both the directionality of experimental heating and potential existence of natural thermal gradients *in ovo* matter; these should be assessed for clutches in their natural environment to better understand embryo behavior and inform future experimental designs.

### Future directions

The discovery of heat-induced hatching opens new questions; for example, what mechanisms enable this response? In turtles, thermoregulatory behavior in *ovo* is enabled by molecular thermal sensors that allow embryos to detect thermal gradients (Ye et al. 2021). It would be worth testing if *A. callidryas* embryos have homologous or convergent thermal sensors and, if so, when in development they are expressed. Moreover, the proof-of-concept demonstration of an embryo VT_Max_ in one frog raises the question of how widespread this is, motivating comparative research on thermosensitive embryo behavior across anurans. It would be particularly interesting to compare species with different oviposition strategies (e.g., clutches with different amounts of jelly, with/without leaf-wrapping, and with/without parental care) to assess how this variation affects the thermo-hydric environment of embryos and their behavioral responses to warming. This could help elucidate which species are more vulnerable to increasing temperature and desiccation risk as climate change continues.

## Supplementary Material

obac041_Supplemental_File

## References

[bib1] Akaike H . 1973. Maximum likelihood identification of Gaussian autoregressive moving average models. Biometrika 60:255–65.

[bib2] Allan RP , Soden BJ. 2008. Atmospheric warming and the amplification of precipitation extremes. Science 321: 1481–4. 18687921 10.1126/science.1160787

[bib3] Anderson RC , Andrade DV. 2017. Trading heat and hops for water: dehydration effects on locomotor performance, thermal limits, and thermoregulatory behavior of a terrestrial toad. Ecol Evol 7:9066–75. 29152198 10.1002/ece3.3219 PMC5677477

[bib4] Bates D , Maechler M, Bolker B, Walker S. 2015. Fitting linear mixed-effects models using lme4. J Stat Softw 67:1–48.

[bib5] Bozdogan H . 2000. Akaike's information criterion and recent developments in information complexity. J Math Psychol 44:62–91. 10733858 10.1006/jmps.1999.1277

[bib6] Bradford DF . 1990. Incubation time and rate of embryonic development in amphibians: the influence of ovum size, temperature, and reproductive mode. Physiol Zool 63:1157–80.

[bib7] Cadena V , Tattersall GJ. 2009. The effect of thermal quality on the thermoregulatory behavior of the bearded dragon *Pogona vitticeps*: influences of methodological assessment. Physiol Biochem Zool 82:203–17. 19323642 10.1086/597483

[bib8] Camacho A , Rusch T, Ray G, Telemeco RS, Rodrigues MT, Angilletta MJ. 2018. Measuring behavioral thermal tolerance to address hot topics in ecology, evolution, and conservation. J Therm Biol 73:71–9. 29549993 10.1016/j.jtherbio.2018.01.009

[bib9] Collin R , Rebolledo AP, Smith E, Chan KYK. 2021. Thermal tolerance of early development predicts the realized thermal niche in marine ectotherms. Funct Ecol 35, 1679–92.

[bib10] Cowles RB , Bogert CM. 1944. A preliminary study of the thermal requirements of desert reptiles. Bull Am Mus Nat 83:261–96.

[bib11] Dai A . 2012. Increasing drought under global warming in observations and models. Nat Clim Chang 3:52.

[bib12] Delia JR , Ramírez-Bautista A, Summers K. 2014. Glassfrog embryos hatch early after parental desertion. Proc. Royal Soc. B 281:20133237. 10.1098/rspb.2013.3237 PMC4024282 24789892

[bib13] Delia J , Bravo-Valencia L, Warkentin KM. 2017. Patterns of parental care in Neotropical glassfrogs: fieldwork alters hypotheses of sex-role evolution. J Evol Biol 30:898–914. 28241390 10.1111/jeb.13059

[bib14] Delia J , Bravo-Valencia L, Warkentin KM. 2020. The evolution of extended parental care in glassfrogs: do egg-clutch phenotypes mediate coevolution between the sexes? Ecol Monogr 90:e01411.

[bib19] Díaz-Ricaurte JC , Guevara-Molina EC, Alves-Nunes JM, Serrano FC, Hrncir M. 2022a. Linking body condition and thermal physiology in limping crickets: does limb autotomy incur costs concerning behavioral thermal tolerance? J Exp Zool A: Ecol Integr Physiol 337:393−402. 35167191 10.1002/jez.2577

[bib15] Díaz-Ricaurte JC , Serrano FC. 2020. It is getting hot in here: behavioural thermal tolerance of *Amphisbaena alba* (Squamata: Amphisbaenidae). Herpetol Notes 13:101–3.

[bib17] Díaz-Ricaurte JC , Serrano FC. 2021. Short-term captivity does not affect immediate voluntary thermal maximum of a neotropical pitviper: implications for behavioral thermoregulation. J Exp Zool A: Ecol Integr Physiol 335:199–206. 33258560 10.1002/jez.2433

[bib16] Díaz-Ricaurte JC , Serrano FC, Guevara-Molina EC, Araujo C, Martins M. 2020. Does behavioral thermal tolerance predict distribution pattern and habitat use in two sympatric Neotropical frogs? PLoS One 15:e0239485. 32960914 10.1371/journal.pone.0239485 PMC7508379

[bib18] Díaz-Ricaurte JC , Serrano FC, Martins M. 2022b. VTMaxHerp: a data set of voluntary thermal maximum temperatures of amphibians and reptiles from two Brazilian hotspots. Ecology 103:e3602. 34897661 10.1002/ecy.3602

[bib20] Donnelly MA , Crump ML. 1998. Potential effects of climate change on two neotropical amphibian assemblages. Clim Change 39:541–61.

[bib21] Duarte H , Tejedo M, Katzenberger M, Marangoni F, Baldo D, Beltrán JF, González-Voyer A. 2012. Can amphibians take the heat? Vulnerability to climate warming in subtropical and temperate larval amphibian communities. Global Change Biol 18:412–21.

[bib22] Eme J , Mueller CA, Manzon RG, Somers CM, Boreham DR, Wilson JY. 2015. Critical windows in embryonic development: shifting incubation temperatures alter heart rate and oxygen consumption of Lake Whitefish (*Coregonus clupeaformis*) embryos and hatchlings. Comp Biochem Physiol A 179:71–80. 10.1016/j.cbpa.2014.09.005 25236178

[bib23] Frederich M , Pörtner HO. 2000. Oxygen limitation of thermal tolerance defined by cardiac and ventilatory performance in spider crab, *Maja squinado**.* Am J Physiol Regul Integr Comp Physiol 279:R1531–8. 11049833 10.1152/ajpregu.2000.279.5.R1531

[bib24] Gonzáles K , Warkentin KM, Güell BA. 2021. Dehydration-induced mortality and premature hatching in gliding treefrogs with even small reductions in humidity. Ichthyol Herpetol 109:21–30.

[bib25] Guevara-Molina EC , Gomes FR, Camacho Guerrero A. 2020. Effects of dehydration on thermoregulatory behavior and thermal tolerance limits of *Rana catesbeiana* (Anura: Ranidae). J Therm Biol 93:102721. 33077134 10.1016/j.jtherbio.2020.102721

[bib26] Güell BA , Warkentin KM. 2018. When and where to hatch? Red-eyed treefrog embryos use light cues in two contexts. PeerJ 6:e6018. 30533307 10.7717/peerj.6018 PMC6283037

[bib27] Harkey GA , Semlitsch RD. 1988. Effects of temperature on growth, development, and color polymorphism in the ornate chorus frog *Pseudacris ornata*. Copeia 1988:1001–7.

[bib28] Hoffmann EP , Cavanough KL, Mitchell NJ. 2021. Low desiccation and thermal tolerance constrains a terrestrial amphibian to a rare and disappearing microclimate niche. Conserv Physiol 9:coab027. 33959292 10.1093/conphys/coab027 PMC8084025

[bib29] Ji X , Du WG. 2001. The effects of thermal and hydric environments on hatching success, embryonic use of energy and hatchling traits in a colubrid snake, *Elaphe carinata*. Comp Biochem Physiol A: Mol Integr Physiol 129:461–71. 11423316 10.1016/s1095-6433(01)00271-9

[bib30] Jung J , Serrano-Rojas SJ, Warkentin KM. 2020. Multimodal mechanosensing enables treefrog embryos to escape egg predators. J Exp Biol 223:jeb236141. 33188064 10.1242/jeb.236141

[bib31] Kuramoto M . 1975. Embryonic temperature adaptation in development rate of frogs. Physiol Zool 48:360–6.

[bib32] Lips KR , Burrowes PA, Mendelson JR, Parra Olea G. 2005. Amphibian population declines in Latin América: a synthesis. Biotropica 37:222–6.

[bib33] Lips KR , Diffendorfer J, Mendelson JR III, Sears MV. 2008. Riding the wave: reconciling the roles of disease and climate change in amphibian declines. PLoS Biol 6:e72. 18366257 10.1371/journal.pbio.0060072 PMC2270328

[bib34] Lutterschmidt WI , Hutchison VH. 1997. The critical thermal maximum: history and critique. Can J Zool 75:1561–74.

[bib35] McLaren IA , Cooley JM. 1972. Temperature adaptation of embryonic development rate among frogs. Physiol Zool 45:223–8.

[bib36] Mendez-Narvaez J , Flechas SV, Amezquita A. 2015. Foam nests provide context-dependent thermal insulation to embryos of three leptodactylid frogs. Physiol Biochem Zool 88:246–53. 25860824 10.1086/680383

[bib37] Méndez-Narváez J , Warkentin KM. 2022. Reproductive colonization of land by frogs: embryos and larvae excrete urea to avoid ammonia toxicity. Ecol Evol 12:e8570. 35222954 10.1002/ece3.8570 PMC8843769

[bib38] Miaud C , Dejean T, Savard K, Millery-Vigues A, Valentini A, Gaudin NCG, Garner TW. 2016. Invasive North American bullfrogs transmit lethal fungus *Batrachochytrium dendrobatidis* infections to native amphibian host species. Biol Invasions 18:2299–308.

[bib39] Mitchell NJ . 2002. Low tolerance of embryonic desiccation in the terrestrial nesting frog *Bryobatrachus nimbus* (Anura: Myobatrachinae). Copeia 2002:364–73.

[bib40] Mitchell A , Bergmann PJ. 2016. Thermal and moisture habitat preferences do not maximize jumping performance in frogs. Funct Ecol 30:733–42.

[bib41] Mitchell NJ , Seymour RS. 2000. Effects of temperature on the energy cost and timing of embryonic and larval development of the terrestrially breeding moss frog, *Bryobatrachus nimbus*. Physiol Biochem Zool 73:829–40. 11121356 10.1086/318097

[bib45] Mueller CA , Bucsky J, Korito L, Manzanares S. 2019. Immediate and persistent effects of temperature on oxygen consumption and thermal tolerance in embryos and larvae of the Baja California chorus frog, *Pseudacris hypochondriaca*. Front Physiol 10:754. 31275167 10.3389/fphys.2019.00754 PMC6591441

[bib43] Mueller CA , Eme J, Burggren WW, Rundle SD, Roghair RD. 2015a. Challenges and opportunities in integrative developmental physiology. Comp Biochem Physiol A 184:113–24. 10.1016/j.cbpa.2015.02.013 PMC4646063 25711780

[bib44] Mueller CA , Eme J, Manzon RG, Somers CM, Boreham DR, Wilson JY. 2015b. Embryonic critical windows: changes in incubation temperature alter survival, hatchling phenotype and cost of development in Lake whitefish (*Coregonus clupeaformis*). J Comp Physiol B 185:315–31. 25585944 10.1007/s00360-015-0886-8

[bib42] Mueller CA , Joss JMP, Seymour RS. 2011. The energy cost of embryonic development in fishes and amphibians, with emphasis on new data from the Australian lungfish, *Neoceratodus forsteri*. J Comp Physiol B 181:43–52. 20676654 10.1007/s00360-010-0501-y

[bib47] Navas CA , Carvajalino-Fernández JM, Saboyá-Acosta LP, Rueda-Solano LA, Carvajalino-Fernández MA. 2013. The body temperature of active amphibians along a tropical elevation gradient: patterns of mean and variance and inference from environmental data. Funct Ecol 27:1145–54.

[bib46] Navas CA , Gomes FR, Carvalho JE. 2008. Thermal relationships and exercise physiology in anuran amphibians: integration and evolutionary implications. Comp Biochem Physiol 151:344–62. 10.1016/j.cbpa.2007.07.003 17703978

[bib48] Ojanguren AF , Brana F. 2003. Thermal dependence of embryonic growth and development in brown trout. J Fish Biol 62:580–90.

[bib49] Parmesan C . 2007. Influences of species, latitudes and methodologies on estimates of phenological response to global warming. Global Change Biol 13:1860–72.

[bib50] Pounds JA , Bustamante MR, Coloma LA, Consuegra JA, Fogden MP, Foster PN, Ron S. 2006. Widespread amphibian extinction from epidemic disease driven by global warming. Nature 439:161–7. 16407945 10.1038/nature04246

[bib51] Pyburn WF . 1970. Breeding behavior of the leaf-frogs *Phyllomedusa callidryas* and *Phyllomedusa dacnicolor* in Mexico. Copeia 1970:209–18.

[bib52] R Core Team . 2020. R: a language and environment for statistical computing. Vienna: R Foundation for Statistical Computing. http://www.R-project.org/.

[bib53] Rogge JR , Warkentin KM. 2008. External gills and adaptive embryo behavior facilitate synchronous development and hatching plasticity under respiratory constraint. J Exp Biol 211:3627–35. 18978228 10.1242/jeb.020958

[bib54] Rudin-Bitterli TS , Evans JP, Mitchell NJ. 2020. Geographic variation in adult and embryonic desiccation tolerance in a terrestrial-breeding frog. Evolution 74:1186–99. 32255513 10.1111/evo.13973

[bib56] Salica MJ , Vonesh JR, Warkentin KM. 2017. Egg clutch dehydration induces early hatching in red-eyed treefrogs, *Agalychnis callidryas*. PeerJ 5:e3549. 28717595 10.7717/peerj.3549 PMC5511700

[bib57] Salthe SN . 1965. Increase in volume of the perivitelline chamber during development of *Rana pipiens* Schreber. Physiol Zool 38:80–98.

[bib58] Schneider-Maunoury L , Lefebvre V, Ewers RM, Medina-Rangel GF, Peres CA, Somarriba E, Urbina-Cardona N, Pfeifer M. 2016. Abundance signals of amphibians and reptiles indicate strong edge effects in Neotropical fragmented forest landscapes. Biol Conserv 200:207–15.

[bib59] Smith C , Telemeco RS, Angilletta MJ, VandenBrooks JM. 2015. Oxygen supply limits the heat tolerance of lizard embryos. Biol Lett 11:20150113. 25926695 10.1098/rsbl.2015.0113 PMC4424623

[bib61] Stuart SN , Chanson JS, Cox NA, Young BE, Rodrigues AS, Fischman DL, Waller RW. 2004. Status and trends of amphibian declines and extinctions worldwide. Science 306:1783–6. 15486254 10.1126/science.1103538

[bib60] Sunday JM , Bates AE, Kearney MR, Colwell RK, Dulvy NK, Longino JT, Huey RB. 2014. Thermal-safety margins and the necessity of thermoregulatory behavior across latitude and elevation. Proc Natl Acad Sci, 111:5610–5. 24616528 10.1073/pnas.1316145111 PMC3992687

[bib62] Tattersall GJ , Sinclair BJ, Withers PC, Fields PA, Seebacher F, Cooper CE, Maloney SK. 2012. Coping with thermal challenges: physiological adaptations to environmental temperatures. Compr Physiol 2:2151–202. 23723035 10.1002/cphy.c110055

[bib63] Tippett C , Warkentin KM. 2017. How not to die if its too dry: a comparison of spontaneous and dehydration-induced hatching in red-eyed treefrogs. Integr Comp Biol 57:e168.

[bib66] Touchon JC , Urbina J, Warkentin KM. 2011. Habitat-specific constraints on induced hatching in a treefrog with reproductive mode plasticity. Behav Ecol 22:169–75.

[bib64] Touchon JC , Warkentin KM. 2009. Negative synergism of rainfall patterns and predators affects frog egg survival. J Anim Ecol 78:715–23. 19486379 10.1111/j.1365-2656.2009.01548.x

[bib65] Touchon JC , Warkentin KM. 2010. Short and long-term effects of the abiotic egg environment on viability, development and vulnerability to predators of a Neotropical anuran. Funct Ecol 24:566–75.

[bib67] Trenberth KE , Dai A, Van Der Schrier G, Jones PD, Barichivich J, Briffa KR, Sheffield J. 2014. Global warming and changes in drought. Nat Clim Chang 4:17–22.

[bib68] von May R , Catenazzi A, Corl A, Santa-Cruz R, Carnaval AC, Moritz C. 2017. Divergence of thermal physiological traits in terrestrial breeding frogs along a tropical elevational gradient. Ecol Evol 7:3257–67. 28480023 10.1002/ece3.2929 PMC5415528

[bib69] Vorsatz LD , Mostert BP, McQuaid CD, Cannicci S, Porri F. 2021. Thermal sensitivity in dual-breathing ectotherms: Embryos and mothers determine species' vulnerability to climate change. Limnol Oceanogr Lett 7:251–60.

[bib70] Wang Y , Qun L. 2006. Comparison of Akaike information criterion (AIC) and Bayesian information criterion (BIC) in selection of stock–recruitment relationships. Fish Res 77:220–5.

[bib71] Warkentin KM . 1995. Adaptive plasticity in hatching age: a response to predation risk trade-offs. Proc Natl Acad Sci 92:3507–10. 11607529 10.1073/pnas.92.8.3507 PMC42196

[bib72] Warkentin KM . 2000. Wasp predation and wasp-induced hatching of red-eyed treefrog eggs. Anim Behav 60:503–10. 11032653 10.1006/anbe.2000.1508

[bib73] Warkentin KM . 2002. Hatching timing, oxygen availability, and external gill regression in the treefrog, *Agalychnis callidryas*. Physiol Biochem Zool 75:155–64. 12024291 10.1086/339214

[bib74] Warkentin KM . 2011a. Environmentally cued hatching across taxa: embryos respond to risk and opportunity. Integr Comp Biol 51:14–25. 21705799 10.1093/icb/icr017

[bib75] Warkentin KM . 2011b. Plasticity of hatching in amphibians: evolution, trade-offs, cues and mechanisms. Integr Comp Biol 51:111–27. 21642239 10.1093/icb/icr046

[bib76] Warkentin KM . 2017. Development of red-eyed treefrog embryos: a staging table for integrative research on environmentally cued hatching. Integr Comp Biol 57:e175.

[bib77] Warkentin KM , Currie CR, Rehner SA. 2001. Egg-killing fungus induces early hatching of red-eyed treefrog eggs. Ecology 82:2860–9.

[bib79] Warkentin KM , Caldwell MS. 2009. Assessing risk: embryos, information, and escape hatching. In: Dukas R, Ratcliffe JM, editors. Cognitive ecology II. Chicago (IL): University of Chicago Press. p. 177–200.

[bib80] Warkentin KM , Cuccaro Diaz J, Güell BA, Jung J, Kim SJ, Cohen KL. 2017. Developmental onset of escape-hatching responses in red-eyed treefrogs depends on cue type. Anim Behav 129:103–12.

[bib78] Warkentin KM , Gomez-Mestre I, McDaniel JG. 2005. Development, surface exposure, and embryo behavior affect oxygen levels in eggs of the red-eyed treefrog, *Agalychnis callidryas*. Physiol Biochem Zool 78:956–66. 16228935 10.1086/432849

[bib81] Wolcott TG , Wolcott DL. 2001. Role of behavior in meeting osmotic challenges. Am Zool 41:795–806.

[bib82] Wygoda ML , Williams AA. 1991. Body temperature in free-ranging tree frogs (*Hyla cinerea*): a comparison with “typical” frogs. Herpetologica 47:328–35.

[bib83] Ye YZ , Zhang H, Li J, Lai R, Yang S, Du WG. 2021. Molecular sensors for temperature detection during behavioral thermoregulation in turtle embryos. Curr Biol 31:2995–3003. 34015251 10.1016/j.cub.2021.04.054

[bib84] Zhao B , Li T, Shine R, Du WG. 2013. Turtle embryos move to optimal thermal environments within the egg. Biol Lett 9:20130337. 23760168 10.1098/rsbl.2013.0337 PMC3730644

